# A fractional complex network model for novel corona virus in China

**DOI:** 10.1186/s13662-020-03182-y

**Published:** 2021-01-06

**Authors:** H. A. A. El-Saka, I. Obaya, H. N. Agiza

**Affiliations:** 1 Mathematics Department, Faculty of Science, Damietta University, New Damietta, 34517 Egypt; 2 Basic Science Department, Nile Higher Institute for Engineering & Technology, Mansoura, Egypt; 3 Department of Mathematics, Faculty of Science, Mansoura University, P.O. Box 64, Mansoura, 35516 Egypt

**Keywords:** Complex networks, Novel coronavirus (COVID-19), Stability analysis, Basic reproductive number and fractional calculus

## Abstract

As is well known the novel coronavirus (COVID-19) is a zoonotic virus and our model is concerned with the effect of the zoonotic source of the coronavirus during the outbreak in China. We present a SEIS complex network epidemic model for the novel coronavirus. Our model is presented in fractional form and with varying population. The steady states and the basic reproductive number are calculated. We also present some numerical examples and the sensitivity analysis of the basic reproductive number for the parameters.

## Introduction

The corona virus family continues to clone new strains that threaten human life. The world is witnessing these days the emergence of a new strain of corona virus in the Chinese city of Wuhan. This novel corona virus strain (COVID-19) is the seventh of the corona family, which includes, for example, influenza, SARS, and Middle East respiratory syndrome (MERS). The family of coronaviruses is characterized by being common among different types of animal species, such as bats, cats, camels, and cattle.

In December 31, 2019, the Chinese city of Wuhan announced the outbreak of a new strain of coronavirus. And this new strain is considered among the zoonotic viruses that are transmitted from animal to human and then transmitted from human to human [1–6].

On February 27, 2020 (at the time of writing), there were 82,294 infected cases of this virus worldwide, according to the World Health Organization’s report No. 38 on the epidemiological situation of the virus, including 2747 deaths in China and 57 death cases outside China [7]. This is equivalent to 3.41% mortality worldwide.

The Chinese city of Wuhan in Hubei Province is the origin of this virus, which quickly spread to many Chinese cities (34 cities so far). Then it moved out of the Chinese territory until it reached 46 countries worldwide [7]. Nowadays, we find that China is close to eradicating the COVID-19 epidemic.

The Chinese city of Wuhan is among the most important cities in China in terms of combining many transportation lines between Chinese cities as well as internal transportation outside China. Wuhan also contains a large market for seafood and animals, which is the source of the emergence and spread of COVID-19.

And when looking at how COVID-19 spreads from person to person, we find that the pattern of spread is not known yet, but most of the current information about the method of spread is based on previous information on corona viruses. Also, the spread of COVID-19 from a person infected with the virus to a healthy person needs close communication with the infected person where there will be an effect of cough and sneezing droplets. It turned out from the current cases of infection, whether simple or severe, that symptoms of this disease (COVID-19) appear in the form of fever, shortness of breath and cough. To date, there is no vaccine for this virus, so general prevention instructions such as avoiding direct contact with infected people and using gloves and face masks should be adhered to.

The study focused in this model on the zoonotic nature of the virus because of its continuous effect on the spread of the virus, especially at the beginning of the spread. In addition, the model was placed in a fractional form, with the community being represented by a heterogeneous network, in order for the model to be more realistic.

In the following section we present a heterogeneous network epidemic model for COVID-19 in a fractional form [8–10] using the Caputo definition. The SEIS scenario was chosen as the mode of diffusion, as it was considered more suitable than the SEIR because some cases have been confirmed to be re-infected with COVID-19 [11–13]. For more information about the basics of fractional calculus and fractional model stability, see [14–20] and [21–24], for networks see [25]. In Sect. 2 we described the model. In Sect. 3 we find the steady states and the basic reproductive function. In Sect. 4 we proved the local stability of the steady states. In Sect. 5 we present the sensitivity analysis to get the most effective parameter and some numerical examples. Section 6 is the conclusion.

## Fractional SEIS model description

In this model we divided the population into three compartments susceptible, exposed and infected. The susceptible individuals can be exposed because of being in close contact with infected one. Also, the infection could be transmitted to a susceptible individual from a zoonotic source of COVID-19 (an unknown animal embracing the virus). This interaction between susceptible and the zoonotic source happened in a homogeneous pattern during buying and walking in the seafood market. Also, the number of zoonotic sources is considered to be constant in the seafood market (sellers put other animals after the animals that were sold). The exposed individual become infected after the incubation period. The infected individual became susceptible again after the infectious period. The city’s population (Wuhan city) is changing as a result of traveling continuously to and from the city. In this model we ignored the births and the deaths.

**Figure 1:**
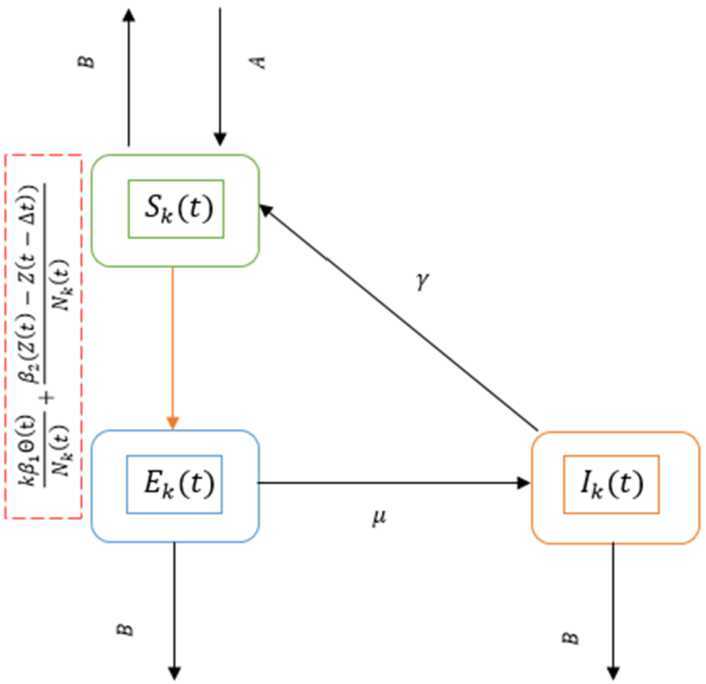
The dynamical interacting of system (2.1)

According to the above system dynamic description, the model is defined as

2.1 $$\begin{aligned}& {}_{0}^{C} D_{t}^{\alpha } S_{k} (t) =A- \frac{k \beta _{1} S_{k} ( t ) \Theta ( t )}{N_{k} ( t )} - \frac{\beta _{2} S_{k} ( t ) (Z ( t ) -Z ( t-\Delta t ) )}{N_{k} ( t )} +\gamma I_{k} ( t ) -B S_{k} ( t ), \\& {}_{0}^{C} D_{t}^{\alpha } E_{k} (t) = \frac{k \beta _{1} S_{k} ( t ) \Theta ( t )}{N_{k} ( t )} + \frac{\beta _{2} S_{k} ( t ) (Z ( t ) -Z ( t-\Delta t ) )}{N_{k} ( t )} -\mu E_{k} ( t ) -B E_{k} ( t ), \\& {}_{0}^{C} D_{t}^{\alpha } I_{k} (t) = \mu E_{k} ( t ) -\gamma I_{k} ( t ) -B I_{k} ( t ), \end{aligned}$$

where *k* is the degree of the node, $1\leq k\leq n$, *n* is the maximum degree of a node. $\Theta ( t )$ is the probability to be linked with an infected node and defined as

$$ \Theta ( t ) = \frac{\sum_{k} kP(k) I_{k} ( t )}{ \langle k \rangle }, $$

where $\langle k \rangle = \sum_{k} kP(k) $.

$P(k)$ is the degree distribution of the population. $N_{k} ( t )$ is the total population of degree *k*. $(Z ( t ) -Z ( t-\Delta t ) )$ is a Heaviside function representing the zoonotic infection force. This function affects only before seafood market closure (from 1 December 2019 to 31 December 2019). After the seafood market closure on 1 January 2020 this function is equal to 0. Other parameters are described in Table 1. We used the Caputo definition for the fractional order $\alpha \in ( 0,1 ]$, which is defined as follows:

$$ {}_{a}^{C} D_{t}^{\alpha } f ( t ) = \frac{1}{\Gamma (1- \alpha )} \int _{a}^{t} ( t - s )^{- \alpha } f' ( s )\,ds. $$

**Table 1:** Parameters description

Parameter	Description
$\beta _{1}$	The infectious rate from infected individual.
$\beta _{2}$	The infectious rate from the zoonotic source.
*μ*	Rate of becoming infected.
*γ*	Recovery rate and be susceptible again.
*A*	The average number of passengers coming into the city.
*B*	Traveling rate from the city.

## Steady states and the basic reproductive number

Let $\mathfrak{S}= \{ ( S_{k} ( t ), E_{k} ( t ), I_{k} ( t ) ) \in R_{+}^{3k},k=1,2,\dots ,n \vert N_{k} ( t ) = S_{k} ( t ) + E_{k} ( t ) + I_{k} ( t ) \leq \frac{A}{B} \} $ be a closed positive invariant set for system (2.1).

We will find the equilibrium points of system (2.1) by putting its equations equal to zero as follows:

$$\begin{aligned}& A- \frac{k \beta _{1} S_{k} ( t ) \Theta ( t )}{N_{k} ( t )} - \frac{\beta _{2} S_{k} ( t ) z(t)}{N_{k} ( t )} +\gamma I_{k} ( t ) - B S_{k} ( t ) =0, \\& \frac{k \beta _{1} S_{k} ( t ) \Theta ( t )}{N_{k} ( t )} + \frac{\beta _{2} S_{k} ( t ) z(t)}{N_{k} ( t )} -\mu E_{k} ( t ) - B E_{k} ( t ) =0, \\& \mu E_{k} ( t ) -\gamma I_{k} ( t ) - B I_{k} ( t ) =0, \end{aligned}$$

where $Z ( t ) -Z ( t-\Delta t ) =z(t)$. It is obvious that system (2.1) has a unique free disease equilibrium point,

$$ P_{0} = \biggl\{ \frac{A}{B},0,0 \biggr\} _{1\leq k\leq n}, $$

with respect to $z ( t ) =0$ and an endemic point $P_{2} = \{ S_{k}^{**}, E_{k}^{**}, I_{k}^{**} \} _{1\leq k\leq n}$, where

$$\begin{aligned}& S_{k}^{**} = \frac{A^{2} ( \mu + B ) ( \gamma + B )}{B [ ( k \beta _{1} \Theta ^{*} + \beta _{2} z ( t ) ) B ( B+ \gamma +\mu ) + A ( \mu + B ) ( \gamma + B ) ]}, \\& E_{k}^{**} = \frac{ ( \gamma + B ) A ( k \beta _{1} \Theta ^{*} + \beta _{2} z ( t ) )}{ ( k \beta _{1} \Theta ^{*} + \beta _{2} z ( t ) ) B ( B+ \gamma +\mu ) + A ( \mu + B ) ( \gamma + B )}, \\& I_{k}^{**} = \frac{\mu A ( k \beta _{1} \Theta ^{*} + \beta _{2} z ( t ) )}{ ( k \beta _{1} \Theta ^{*} + \beta _{2} z ( t ) ) B ( B+ \gamma +\mu ) + A ( \mu + B ) ( \gamma + B )}. \end{aligned}$$

The value of the endemic point changes with respect to the existence of $z ( t )$. If $z ( t ) =0$, then the endemic point take the form $P_{1} = \{ S_{k}^{*}, E_{k}^{*}, I_{k}^{*} \} _{1\leq k\leq n}$, where

$$\begin{aligned}& S_{k}^{*} = \frac{A^{2} ( \mu + B ) ( \gamma + B )}{B [ k \beta _{1} \Theta ^{*} B ( B+ \gamma +\mu ) + A ( \mu + B ) ( \gamma + B ) ]}, \\& E_{k}^{*} = \frac{ ( \gamma + B ) A k \beta _{1} \Theta ^{*}}{k \beta _{1} \Theta ^{*} B ( B+ \gamma +\mu ) + A ( \mu + B ) ( \gamma + B )}, \\& I_{k}^{*} = \frac{\mu Ak \beta _{1} \Theta ^{*}}{k \beta _{1} \Theta ^{*} B ( B+ \gamma +\mu ) + A ( \mu + B ) ( \gamma + B )}. \end{aligned}$$

### The existence of the endemic point

By substituting with the value of $I_{k}^{**}$ into the definition of $\Theta ( t )$ we get the self-consistency equation

$$ \Theta = \frac{1}{ \langle k \rangle } \sum_{k} kp(k) \frac{\mu A ( k \beta _{1} \Theta + \beta _{2} z ( t ) )}{ ( k \beta _{1} \Theta + \beta _{2} z ( t ) ) B ( B+ \gamma +\mu ) + A ( \mu + B ) ( \gamma + B )}. $$

We can put it in the following form:

$$ g ( \Theta ) = \frac{1}{ \langle k \rangle } \sum_{k} kp(k) \frac{\mu A ( k \beta _{1} \Theta + \beta _{2} z ( t ) )}{ ( k \beta _{1} \Theta + \beta _{2} z ( t ) ) B ( B+ \gamma +\mu ) + A ( \mu + B ) ( \gamma + B )} - \Theta =0. $$

Now, we need to get a solution for $g ( \Theta )$ in the interval $\Theta \in (0,1)$. By calculating the value of $g ( \Theta )$ at both 0 and 1 we get

$$ g ( 1 ) = \frac{1}{ \langle k \rangle } \sum_{k} kp(k) \frac{\mu A ( k \beta _{1} + \beta _{2} z ( t ) )}{ ( k \beta _{1} + \beta _{2} z ( t ) ) B ( B+ \gamma +\mu ) + A ( \mu + B ) ( \gamma + B )} - 1< 0 $$

and

$$ g ( 0 ) = \frac{1}{ \langle k \rangle } \sum_{k} kp(k) \frac{\mu A \beta _{2} z ( t )}{\beta _{2} z ( t ) B ( B+ \gamma +\mu ) + A ( \mu + B ) ( \gamma + B )} \geq 0. $$

Therefore, we have two cases.

*Case* 1: If $z ( t )$ exists, then $g ( 0 ) >0$. This leads to the function $g ( \Theta )$ always having a non-trivial solution in the interval $(0,1)$.

*Case* 2: If $z ( t ) =0$, then $g ( 0 ) =0$. Hence, the function $g ( \Theta )$ has a non-trivial solution in the interval $(0,1)$ under the condition

$$ \frac{d g ( \Theta )}{d \Theta } \bigg\vert _{\Theta =0} >0, $$

it follows

3.1 $$ \frac{ \langle k^{2} \rangle }{ \langle k \rangle } \frac{\mu \beta _{1}}{ ( \mu + B ) ( \gamma + B )} >1, $$

where $\langle k^{2} \rangle = \sum_{k} k^{2} p(k)$.

### The basic reproductive number

Only the exposed and infected compartments will be used to find the basic reproductive value [26]. The rate of new infected nodes entering the two compartments $E_{k} (t)$ and $I_{k} ( t )$ is represented by the matrix *F* given by

$$F={\left(\begin{array}{cc} F_{11} & F_{12}\\ F_{21} & F_{22} \end{array}\right)}_{2n\times 2n},$$

where $\mathcal{F}_{11}$, $\mathcal{F}_{12}$, $\mathcal{F}_{21}$ and $\mathcal{F}_{22}$ are $n\times n$ matrices [27]. The following matrix *V* represents the rate of transferring out of and into the two compartments $E_{k} (t)$ and $I_{k} (t)$:

$$V={\left(\begin{array}{cc} V_{11} & V_{12}\\ V_{21} & V_{22} \end{array}\right)}_{2n\times 2n},$$

where $\mathcal{V}_{11}$, $\mathcal{V}_{12}$, $\mathcal{V}_{21}$ and $\mathcal{V}_{22}$ are $n\times n$ matrices. The basic reproductive number is given by the dominant eigenvalue of $F V^{-1}$ calculated at the disease-free equilibrium point $P_{0}$ and $z ( t ) =0$ (pure population). The elements of *F* are given by

$$\begin{array}{c} F_{11}=F_{21}=F_{22}={\left(\begin{array}{cccc} 0 & 0 & \ldots & 0\\ 0 & 0 & \ldots & 0\\ \vdots & \vdots & \ddots & \vdots \\ 0 & 0 & \ldots & 0 \end{array}\right)}_{n\times n},\\ F_{12}=\frac{\beta_{1}}{\langle k\rangle }{\left(\begin{array}{cccc} P(1) & 2P(2) & \ldots & nP(n)\\ 2P(1) & 2^{2}P(2) & \ldots & 2nP(n)\\ \vdots & \vdots & \ddots & \vdots \\ nP(1) & 2nP(2) & \ldots & n^{n}P(n) \end{array}\right)}_{n\times n}, \end{array}$$

and the elements of matrix *V* take the form

$$\begin{array}{c} V_{11}={\left(\begin{array}{cccc} B+\mu & 0 & \ldots & 0\\ 0 & B+\mu & \ldots & 0\\ \vdots & \vdots & \ddots & \vdots \\ 0 & 0 & \ldots & B+\mu \end{array}\right)}_{n\times n},\\ V_{12}={\left(\begin{array}{cccc} 0 & 0 & \ldots & 0\\ 0 & 0 & \ldots & 0\\ \vdots & \vdots & \ddots & \vdots \\ 0 & 0 & \ldots & 0 \end{array}\right)}_{n\times n},\\ V_{21}={\left(\begin{array}{cccc} -\mu & 0 & \ldots & 0\\ 0 & -\mu & \ldots & 0\\ \vdots & \vdots & \ddots & \vdots \\ 0 & 0 & \ldots & -\mu \end{array}\right)}_{n\times n},\\ V_{22}={\left(\begin{array}{cccc} B+\gamma & 0 & \ldots & 0\\ 0 & B+\gamma & \ldots & 0\\ \vdots & \vdots & \ddots & \vdots \\ 0 & 0 & \ldots & B+\gamma \end{array}\right)}_{n\times n}. \end{array}$$

The characteristic equation for the 2*n* eigenvalues *λ* of matrix $F V^{-1}$ is

$$ \lambda ^{n} \biggl( \frac{ \langle k^{2} \rangle }{ \langle k \rangle } \frac{\beta _{1} \mu }{( B +\mu )( B +\gamma )} - \lambda \biggr)^{n} =0, $$

then the basic reproductive number $\mathcal{R}_{0}$ is defined as

$$ \mathcal{R}_{0} = \frac{ \langle k^{2} \rangle }{ \langle k \rangle } \frac{\beta _{1} \mu }{( B +\mu )( B +\gamma )}. $$

#### Theorem 3.1

*Define the basic reproductive number* $\mathcal{R}_{0}$ *as follows*:

$$ \mathcal{R}_{0} = \frac{ \langle k^{2} \rangle }{ \langle k \rangle } \frac{\beta _{1} \mu }{( B +\mu )( B +\gamma )}. $$

1. *If* $z ( t ) =0$ *and* $\mathcal{R}_{0} <1$, *then system* (2.1) *has a unique free disease equilibrium point* $P_{0}$.
2. *If* $z ( t ) =0$ *and* $\mathcal{R}_{0} >1$, *then system* (2.1) *has an unique endemic point* $P_{1}$.
3. *If* $z ( t ) \neq 0$, *then system* (2.1) *always has an endemic point* $P_{2}$.

## Stability analysis of the steady states

### The stability of the free disease equilibrium point $P_{0}$

Firstly, we establish the Jacobian matrix of system (2.1) at $P_{0}$ with respect to $z ( t ) =0$, which takes the form

4.1 $$J(P_{0})={\left(\begin{array}{ccc} C_{11} & C_{12} & C_{13}\\ C_{21} & C_{22} & C_{23}\\ C_{31} & C_{32} & C_{33} \end{array}\right)}_{3n\times 3n},$$

where each sub-matrix $C_{ij}$, $1\leq i$, $j\leq 3$ is an $n\times n$ matrix and is given by

$$\begin{array}{c} C_{11}={\left(\begin{array}{ccc} -B & \cdots & 0\\ \vdots & \ddots & \vdots \\ 0 & \cdots & -B \end{array}\right)}_{n\times n},\\ C_{12}=C_{31}={\left(\begin{array}{ccc} 0 & \cdots & 0\\ \vdots & \ddots & \vdots \\ 0 & \cdots & 0 \end{array}\right)}_{n\times n},\\ C_{13}={\left(\begin{array}{ccc} -\beta_{1}m_{11}+\gamma & \cdots & -\beta_{1}m_{1n}\\ \vdots & \ddots & \vdots \\ -\beta_{1}m_{n1} & \cdots & -\beta_{1}m_{nn}+\gamma \end{array}\right)}_{n\times n},\\ C_{21}={\left(\begin{array}{ccc} 0 & \cdots & 0\\ \vdots & \ddots & \vdots \\ 0 & \cdots & 0 \end{array}\right)}_{n\times n},\\ C_{22}={\left(\begin{array}{ccc} -\mu -B & \cdots & 0\\ \vdots & \ddots & \vdots \\ 0 & \cdots & -\mu -B \end{array}\right)}_{n\times n},\\ C_{23}={\left(\begin{array}{ccc} \beta_{1}m_{11} & \cdots & \beta_{1}m_{1n}\\ \vdots & \ddots & \vdots \\ \beta_{1}m_{n1} & \cdots & \beta_{1}m_{nn} \end{array}\right)}_{n\times n},\\ C_{32}={\left(\begin{array}{ccc} \mu & \cdots & 0\\ \vdots & \ddots & \vdots \\ 0 & \cdots & \mu \end{array}\right)}_{n\times n},\\ C_{33}={\left(\begin{array}{ccc} -\gamma -B & \cdots & 0\\ \vdots & \ddots & \vdots \\ 0 & \cdots & -\gamma -B \end{array}\right)}_{n\times n}, \end{array}$$

where $m_{ij} = \frac{ijp(j)}{ \langle k \rangle }$ $\forall 1\leq i$, $j\leq n$. All eigenvalues of the Jacobian matrix (4.1) should satisfy the following condition:

4.2 $$ \bigl\vert \arg ( x_{i} ) \bigr\vert > \frac{\alpha \pi }{2}. $$

After expanding the Jacobian matrix, we get the following characteristic equation:

4.3 $$ ( B+x )^{n} \bigl( ( \gamma +B+x ) ( \mu +B+x ) \bigr)^{n-1} \biggl( - \mu \beta _{1} \frac{ \langle k^{2} \rangle }{ \langle k \rangle } + ( \gamma +B+x ) ( \mu +B+x ) \biggr) =0. $$

Obviously, we have *n* negative eigenvalues equal to −*B* from the first bracket. From the second bracket we have a second degree equation repeated $n-1$ times in the form

$$ (\gamma +B+x) (\mu +B+x)=0, $$

which having another two negative Eigenvalues $-(\gamma +B)$ and $-(\mu +B)$. Each one is repeated $n-1$ times then we have $2n-2$ negative Eigenvalues from the second bracket. The third bracket in (4.3) is a second degree equation equal to

$$ x^{2} + \rho _{1} x+ \rho _{0} =0, $$

where

$$\begin{aligned}& \rho _{1} =2B+ \gamma + \mu >0, \\& \rho _{0} = \bigl( ( \gamma +B ) ( \mu +B ) \bigr) \biggl( 1- \frac{ \langle k^{2} \rangle }{ \langle k \rangle } \frac{\mu \beta _{1}}{ ( \gamma +B ) ( \mu +B )} \biggr), \\& \rho _{0} = \bigl( ( \gamma +B ) ( \mu +B ) \bigr) ( 1- \mathcal{R}_{0} ). \end{aligned}$$

Therefore, $\rho _{0} >0$; if $\mathcal{R}_{0} <1$ then the third bracket has two negative eigenvalues. Hence condition (4.2) is satisfied.

#### Theorem 4.1

*If* $\mathcal{R}_{0} <1$ *then the free disease steady state* $P_{0}$ *is locally asymptotically stable and unstable if* $\mathcal{R}_{0} >1$.

### The stability of the endemic points

Similarly, forming the Jacobian matrix at the endemic point $P_{2}$ we get

4.4 $$J(P_{2})={\left(\begin{array}{ccc} N_{11} & N_{12} & N_{13}\\ N_{21} & N_{22} & N_{23}\\ N_{31} & N_{32} & N_{33} \end{array}\right)}_{3n\times 3n},$$

where each sub-matrix $N_{ij}$, $1\leq i$, $j\leq 3$ is $n\times n$ matrix and given by

$$\begin{array}{c} N_{11}={\left(\begin{array}{ccc} -(w_{1}+\upsilon_{1})(1-\varepsilon_{1})-B & \cdots & 0\\ \vdots & \ddots & \vdots \\ 0 & \cdots & -(w_{n}+\upsilon_{n})(1-\varepsilon_{n})-B \end{array}\right)}_{n\times n},\\ N_{12}={\left(\begin{array}{ccc} \varepsilon_{1}(w_{1}+\upsilon_{1}) & \cdots & 0\\ \vdots & \ddots & \vdots \\ 0 & \cdots & \varepsilon_{n}(w_{n}+\upsilon_{n}) \end{array}\right)}_{n\times n},\\ N_{13}={\left(\begin{array}{ccc} -\varepsilon_{1}(u_{11}-(w_{1}+\upsilon_{1}))+\gamma & \cdots & -\varepsilon_{1}u_{1n}\\ \vdots & \ddots & \vdots \\ -\varepsilon_{n}u_{n1} & \cdots & -\varepsilon_{n}(u_{nn}-(w_{n}+\upsilon_{n}))+\gamma \end{array}\right)}_{n\times n},\\ N_{21}={\left(\begin{array}{ccc} \left(w_{1}+\upsilon_{1})(1-\varepsilon_{1}\right) & \cdots & 0\\ \vdots & \ddots & \vdots \\ 0 & \cdots & \left(w_{n}+\upsilon_{n})(1-\varepsilon_{n}\right) \end{array}\right)}_{n\times n},\\ N_{22}={\left(\begin{array}{ccc} -\varepsilon_{1}(w_{1}+\upsilon_{1})-\mu -B & \cdots & 0\\ \vdots & \ddots & \vdots \\ 0 & \cdots & -\varepsilon_{n}(w_{n}+\upsilon_{n})-\mu -B \end{array}\right)}_{n\times n},\\ N_{23}={\left(\begin{array}{ccc} \varepsilon_{1}(u_{11}-(w_{1}+\upsilon_{1})) & \cdots & \varepsilon_{1}u_{1n}\\ \vdots & \ddots & \vdots \\ \varepsilon_{n}u_{n1} & \cdots & \varepsilon_{n}(u_{nn}-(w_{n}+\upsilon_{n})) \end{array}\right)}_{n\times n},\\ N_{31}={\left(\begin{array}{ccc} 0 & \cdots & 0\\ \vdots & \ddots & \vdots \\ 0 & \cdots & 0 \end{array}\right)}_{n\times n},\\ N_{32}={\left(\begin{array}{ccc} \mu & \cdots & 0\\ \vdots & \ddots & \vdots \\ 0 & \cdots & \mu \end{array}\right)}_{n\times n},\\ N_{33}={\left(\begin{array}{ccc} -\gamma -B & \cdots & 0\\ \vdots & \ddots & \vdots \\ 0 & \cdots & -\gamma -B \end{array}\right)}_{n\times n}, \end{array}$$

where

$$\begin{aligned}& w_{i} = \frac{\beta _{1} i \Theta ^{*}}{N_{i}^{*}},\qquad \varepsilon _{i} = \frac{S_{i}^{*}}{N_{i}^{*}},\qquad u_{ij} = \frac{\beta _{1} ijp(j)}{ \langle k \rangle }, \\& \upsilon _{i} = \frac{\beta _{2} z ( t )}{N_{i}^{*}},\qquad 1- \varepsilon _{i} >0,\quad \forall 1\leq i,j\leq n. \end{aligned}$$

The characteristic equation has the form

4.5 $$\begin{aligned}& ( B+x )^{n} \prod_{i=1}^{n} \bigl( ( x+ \mu + B ) ( x+ \gamma + B ) + ( w_{i} + \upsilon _{i} ) ( x+ \gamma + B + \mu ) \bigr) \\& \quad {}\times \Biggl( 1- \sum_{i=1}^{n} \frac{\varepsilon _{i} \mu u_{ii}}{ ( ( x+ \mu + B ) ( x+ \gamma + B ) + ( w_{i} + \upsilon _{i} ) ( x+ \gamma + B + \mu ) )} \Biggr) =0. \end{aligned}$$

It is clear that Eq. (4.5) has *n* negative eigenvalues equal to −*B*. The next 2*n* eigenvalues could be obtained from the second part of Eq. (4.5), which is defined as a polynomial function of degree 2*n* as follows:

$$\begin{aligned} \Omega ( x ) =& \prod_{i=1}^{n} \bigl( ( x+ \mu + B ) ( x+ \gamma + B ) + ( w_{i} + \upsilon _{i} ) ( x+ \gamma + B + \mu ) \bigr) \\ &{}\times \Biggl( 1- \sum_{i=1}^{n} \frac{\varepsilon _{i} \mu u_{ii}}{ ( ( x+ \mu + B ) ( x+ \gamma + B ) + ( w_{i} + \upsilon _{i} ) ( x+ \gamma + B + \mu ) )} \Biggr). \end{aligned}$$

Now, we will search for the roots of $\Omega ( x )$ instead of calculating them. In the first case we suppose that

$$ \bigl( ( x+ \mu + B ) ( x+ \gamma + B ) + ( w_{i} + \upsilon _{i} ) ( x+ \gamma + B + \mu ) \bigr) =0, $$

which is an equation of degree two with positive coefficients. That means that we have two negative eigenvalues $- \xi _{i}^{1}$, $-\xi _{i}^{2}$ depending on $w_{i}$ ($w_{i}$ has an increasing value) and having the values

$$\begin{aligned}& \xi _{i}^{1} = \frac{ ( 2B+ \gamma + \mu + w_{i} + \upsilon _{i} )}{2} - \frac{\sqrt{\overline{D}}}{2}, \\& \xi _{i}^{2} = \frac{ ( 2B+ \gamma + \mu + w_{i} + \upsilon _{i} )}{2} + \frac{\sqrt{\overline{D}}}{2}, \end{aligned}$$

where

$$\begin{aligned} \overline{D} =& ( 2B+ \gamma + \mu + w_{i} + \upsilon _{i} )^{2} \\ & {}-4 \bigl( ( \gamma +B ) ( \mu +B ) +(B+ \gamma + \mu ) ( w_{i} + \upsilon _{i} ) \bigr),\quad \xi _{i}^{1}, \xi _{i}^{2} >0 \text{ and } \xi _{i}^{1} < \xi _{i}^{2}, \forall 1\leq i\leq n. \end{aligned}$$

Therefore, we have the last 2*n* negative eigenvalues. In the second case, we suppose

$$\begin{aligned} \Omega ( x ) =& \bigl( ( x+ \mu + B ) ( x+ \gamma + B ) + ( w_{1} + \upsilon _{1} ) ( x+ \gamma + B + \mu ) \bigr) \\ &{}\times \bigl( ( x+ \mu + B ) ( x+ \gamma + B ) + ( w_{2} + \upsilon _{2} ) ( x+ \gamma + B + \mu ) \bigr) \\ &{} \cdots \bigl( ( x+ \mu + B ) ( x+ \gamma + B ) + ( w_{n} + \upsilon _{n} ) ( x+ \gamma + B + \mu ) \bigr) \\ &{} - \varepsilon _{1} \mu u_{11} \bigl( ( x+ \mu + B ) ( x+ \gamma + B ) + ( w_{2} + \upsilon _{2} ) ( x+ \gamma + B + \mu ) \bigr) \\ &{}\times \bigl( ( x+ \mu + B ) ( x+ \gamma + B ) + ( w_{3} + \upsilon _{3} ) ( x+ \gamma + B + \mu ) \bigr) \\ &{} \cdots \bigl( ( x+ \mu + B ) ( x+ \gamma + B ) + ( w_{n} + \upsilon _{n} ) ( x+ \gamma + B + \mu ) \bigr)-\cdots \\ &{} - \varepsilon _{n} \mu u_{nn} \bigl( ( x+ \mu + B ) ( x+ \gamma + B ) + ( w_{1} + \upsilon _{1} ) ( x+ \gamma + B + \mu ) \bigr) \\ &{}\times \bigl( ( x+ \mu + B ) ( x+ \gamma + B ) + ( w_{2} + \upsilon _{2} ) ( x+ \gamma + B + \mu ) \bigr) \\ &{} \cdots \bigl( ( x+ \mu + B ) ( x+ \gamma + B ) + ( w_{n-1} + \upsilon _{n-1} ) ( x+ \gamma + B + \mu ) \bigr) =0, \end{aligned}$$

which is a continuous function. We can put the function $\Omega ( x )$ in a more simple form as follows:

$$\begin{aligned} \Omega ( x ) =& \bigl( \bigl( x+ \xi _{1}^{1} \bigr) \bigl( x+ \xi _{1}^{2} \bigr) \bigl( x+ \xi _{2}^{1} \bigr) \bigl( x+ \xi _{2}^{2} \bigr) \dots \bigl( x+ \xi _{n}^{1} \bigr) \bigl( x+ \xi _{n}^{2} \bigr) \bigr) \\ &{}- \varepsilon _{1} \mu u_{11} \bigl( \bigl( x+ \xi _{2}^{1} \bigr) \bigl( x+ \xi _{2}^{2} \bigr) \bigl( x+ \xi _{3}^{1} \bigr) \bigl( x+ \xi _{3}^{2} \bigr) \dots \bigl( x+ \xi _{n}^{1} \bigr) \bigl( x+ \xi _{n}^{2} \bigr) \bigr) \\ &{}- \varepsilon _{2} \mu u_{22} \bigl( \bigl( x+ \xi _{1}^{1} \bigr) \bigl( x+ \xi _{1}^{2} \bigr) \bigl( x+ \xi _{3}^{1} \bigr) \bigl( x+ \xi _{3}^{2} \bigr) \dots \bigl( x+ \xi _{n}^{1} \bigr) \bigl( x+ \xi _{n}^{2} \bigr) \bigr) -\cdots \\ &{}- \varepsilon _{n} \mu u_{nn} \bigl( \bigl( x+ \xi _{1}^{1} \bigr) \bigl( x+ \xi _{1}^{2} \bigr) \bigl( x+ \xi _{2}^{1} \bigr) \bigl( x+ \xi _{2}^{2} \bigr) \dots \bigl( x+ \xi _{n-1}^{1} \bigr) \bigl( x+ \xi _{n-1}^{2} \bigr) \bigr), \end{aligned}$$

we can observe that

$$ \Omega \bigl( - \xi _{i}^{1} \bigr) \Omega \bigl( - \xi _{i+1}^{1} \bigr) < 0,\quad \forall 1\leq i\leq n, $$

therefore, we have one root in the interval $[- \xi _{i}^{1},- \xi _{i+1}^{1} ]$. In general, we have $n-1$ negative solutions in the interval [$- \xi _{1}^{1},- \xi _{n}^{1} $]. Similarly, with $\xi _{i}^{2}$ we get $n -1$ negative solutions in $[- \xi _{1}^{2},- \xi _{n}^{2} ]$. Searching for the last two roots, we have $\Omega ( - \xi _{1}^{1} ) <0$ and $\Omega ( 0 ) >0$ then we get one more negative solution in the interval $[- \xi _{1}^{1},0]$. Similarly, we can see that $\Omega ( - \xi _{1}^{2} ) <0$. Then we get another negative solution in the interval $[- \xi _{1}^{2},0]$. Finally, the function $\Omega ( x )$ has 2*n* negative solutions in the interval $[- \xi _{n}^{2},0]$. Hence, condition (4.2) is satisfied and the endemic equilibrium point $P_{2}$ is locally asymptotically stable.

#### Theorem 4.2

*The endemic steady state* $P_{2}$ *is always locally asymptotically stable*.

#### Remark 1

When $z ( t ) =0$ and $\mathcal{R}_{0} >1$, then the last proof is valid for $P_{1}$ and it will be locally asymptotically stable.

## Sensitivity analysis and numerical simulation

### Sensitivity of the parameters

Sensitivity analysis shows us which of the parameters used in our mathematical model is the most effective in spreading the infection [28]. In the definition of $\mathcal{R}_{0}$, it is depending on five variables *μ*, $\beta _{1}$, *γ*, *B* and *k̅* where *k̅* is the ratio between the second and the first moment of the node degree *k* as an additional parameter. Using the sensitivity index $\mathcal{S}_{r}^{\mathcal{R}_{0}}$ which mean the sensitivity of the basic reproductive number with respect to *r* (any chosen parameter) with the definition

$$ \mathcal{S}_{r}^{\mathcal{R}_{0}} = \frac{\partial \mathcal{R}_{0}}{\partial r} \frac{r}{\mathcal{R}_{0}}. $$

For example, $\mathcal{S}_{r}^{\mathcal{R}_{0}} =1$ means that any increasing (decreasing) of the value of *r* by *v*% increases (decreases) the value of $\mathcal{R}_{0}$ by the same percentage. In the opposite case, $\mathcal{S}_{r}^{\mathcal{R}_{0}} =-1$ means that any increasing (decreasing) of the value of *r* by *v*% decreases (increases) the value of $\mathcal{R}_{0}$ by the same percentage. After applying the sensitivity analysis, we get the following sensitivity indices:

$$\begin{aligned}& \mathcal{S}_{\mu }^{\mathcal{R}_{0}} = \frac{B}{B + \mu }, \\& \mathcal{S}_{\beta _{1}}^{\mathcal{R}_{0}} =1, \\& \mathcal{S}_{B}^{\mathcal{R}_{0}} =- B \biggl( \frac{1}{B + \gamma } + \frac{1}{B + \mu } \biggr), \\& \mathcal{S}_{\gamma }^{\mathcal{R}_{0}} =- \frac{\gamma }{B + \gamma }, \\& \mathcal{S}_{\overline{k}}^{\mathcal{R}_{0}} =1. \end{aligned}$$

Using the values in Table 2, group 2 we get the following values for the sensitivity indices:

$$\begin{aligned}& \mathcal{S}_{\mu }^{\mathcal{R}_{0}} =1.27,\qquad \mathcal{S}_{\beta _{1}}^{\mathcal{R}_{0}} =1,\qquad \mathcal{S}_{B}^{\mathcal{R}_{0}} =-2.0260, \\& \mathcal{S}_{\gamma }^{\mathcal{R}_{0}} =-3.4140,\qquad \mathcal{S}_{\overline{k}}^{\mathcal{R}_{0}} =1. \end{aligned}$$

It is obvious that the parameter *γ* is the most sensitive parameter i.e. if this parameter increased by 10%, the value of $\mathcal{R}_{0}$ will be decreased by 34.14%. Notice that the values of the sensitivity indices can be changed with respect to the parameters value.

**Table 2:** The value of the parameters used in the model and the initial values

Parameter	Group 1	Group 2
	Value	Value
$\beta _{1}$	0.2	0.6
$\beta _{2}$	0.1	0.1
*μ*	0.5	0.27
*γ*	0.8	0.2
*A*	652,451	652,451
*B*	0.5553431859205776	0.0553431859205776
$N_{0}$	11,081,000	11,081,000
*S*(0)	11,080,139	11,080,139
*E*(0)	820	820
*I*(0)	41	41

### Numerical simulation

In this section, we used an Adams-type predictor-corrector method [19, 20] for solving system (2.1), showing the results obtained in previous sections. We have a BA random scale free network with $p ( k ) =m k^{- \gamma _{1}}$, where *m* is a constant satisfies $\sum_{k} p ( k ) =1$ and $2< \gamma _{1} <3$ is the exponent of the power law distribution. Choosing $\gamma _{1} =2.3$ and $n=100$, we present the following examples.

#### Example 5.1

**Figure 2:**
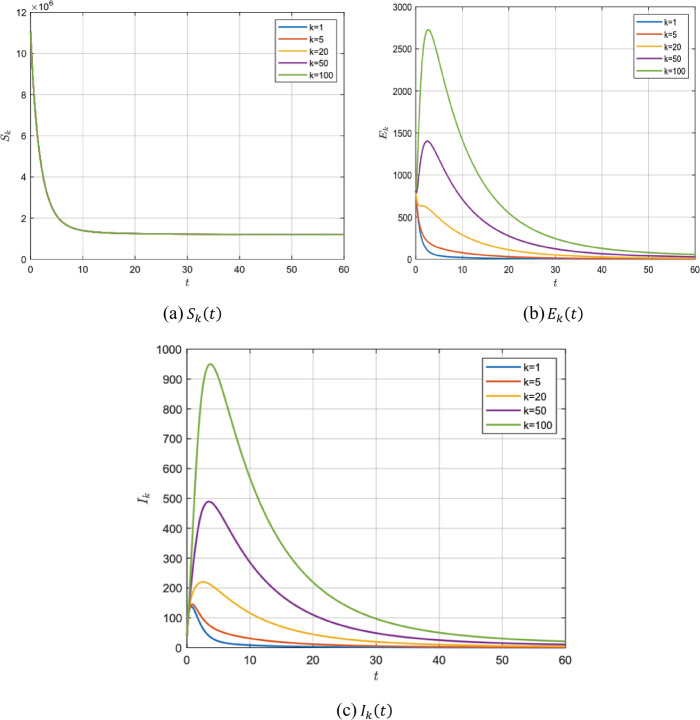
$\alpha =0.95$ and $\mathcal{R}_{0} =0.7953$

**Figure 3:**
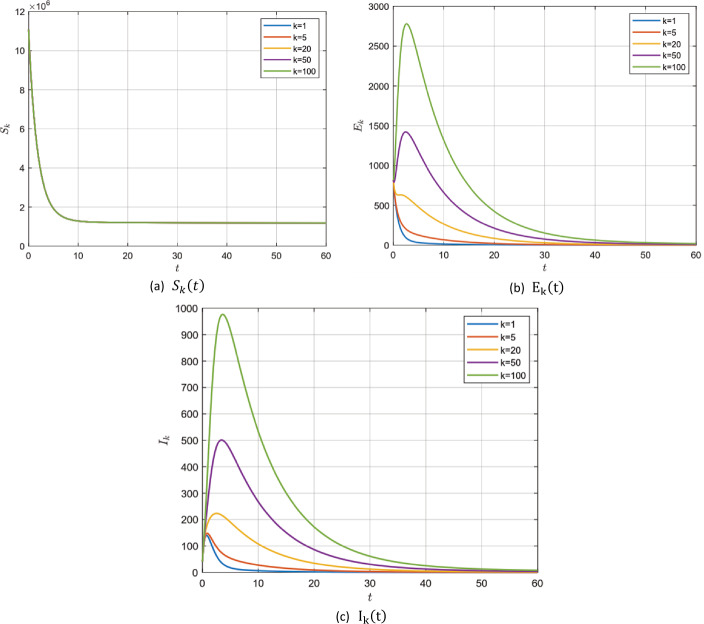
$\alpha =0.98$ and $\mathcal{R}_{0} =0.7953$

**Figure 4:**
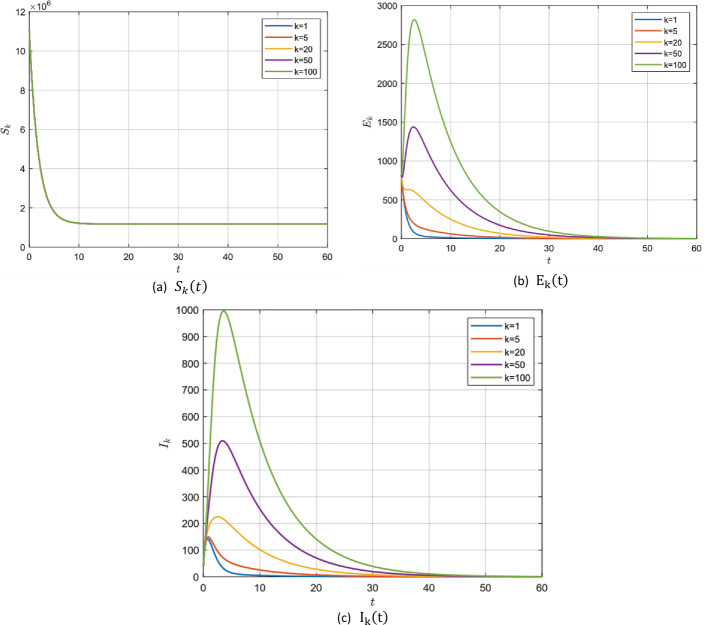
$\alpha =1$ and $\mathcal{R}_{0} =0.7953$

In the absence of the zoonotic effect ($z ( t ) =0$), choosing the values in group 1, Table 2, for model (2.1) parameters we get $\mathcal{R}_{0} =0.7953<1$. In this case, system (2.1) has a unique disease-free steady state $P_{0}$ which is locally asymptotically stable according to Theorem 4.1. It is shown for $S_{k} ( t )$, $E_{k} ( t )$ and $I_{k} ( t )$ for different values of *k* with fractional order $\alpha =0.95, 0.98$ and 1 in Figs. 2–4.

#### Example 5.2

**Figure 5:**
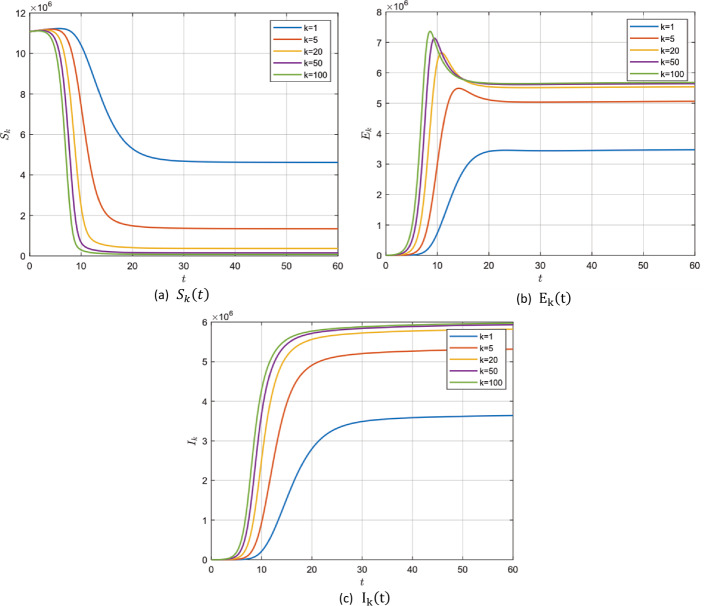
$\alpha =0.95$ and $\mathcal{R}_{0} =22.1121$

**Figure 6:**
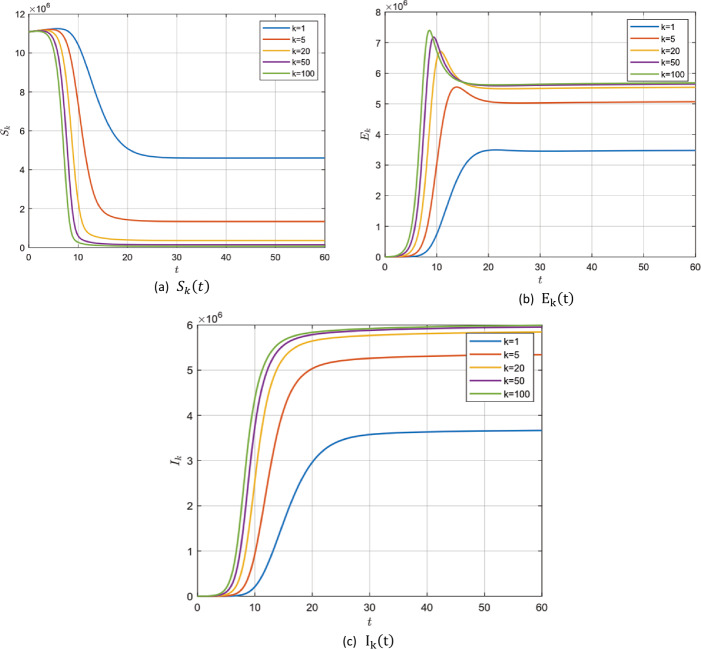
$\alpha =0.98$ and $\mathcal{R}_{0} =22.1121$

**Figure 7:**
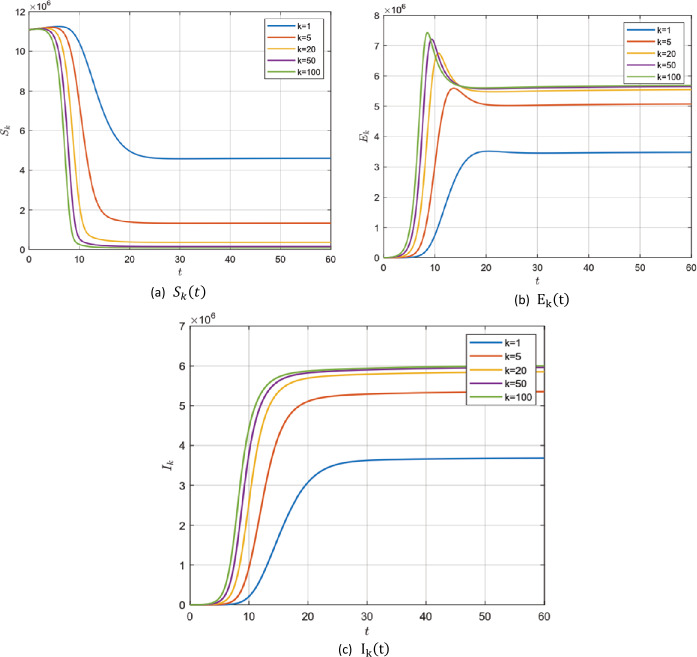
$\alpha =1$ and $\mathcal{R}_{0} =22.1121$

In the absence of the zoonotic effect ($z ( t ) =0$), choosing the values in group 2, Table 2, for model (2.1) parameters we get $\mathcal{R}_{0} =22.1121>1$. In this case, system (2.1) has a unique endemic steady state $P_{1}$ which is locally asymptotically stable according to Remark 1. It is shown for $S_{k} ( t )$, $E_{k} ( t )$ and $I_{k} ( t )$ for different values of *k* with fractional order $\alpha =0.95, 0,98$ and 1 in Fig. 5–7.

#### Example 5.3

**Figure 8:**
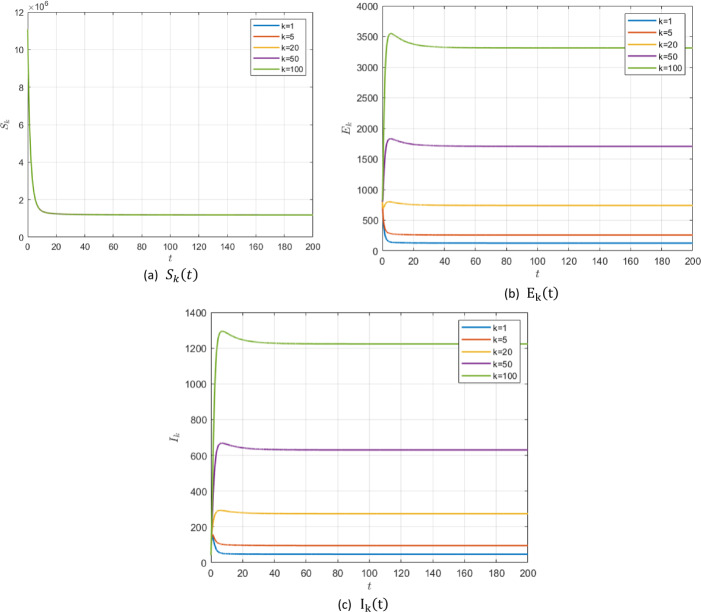
$\alpha =0.95$ and $\mathcal{R}_{0} =0.7953$

In the case of existence of the zoonotic effect ($z ( t ) =1000$ for $\Delta t=100$), choosing the values in group 1, Table 2, for model (2.1) parameters we get $\mathcal{R}_{0} =0.7953<1$. In this case, system (2.1) has a unique endemic steady state $P_{2}$ which is locally asymptotically stable according to Theorem 4.2. It is shown for $S_{k} ( t )$, $E_{k} ( t )$ and $I_{k} ( t )$ for different values of *k* with fractional order $\alpha =0.95$ in Fig. 8.

After the numerical simulation, we can observe the effect of the fractional order *α*. Decreasing the value of *α* gives a larger region for stability to our system i.e. for lower values for *α* the more extended and lower peak we get. It is known that increasing the number of infected individuals in a short period of time can lead to the collapse of the health organization anywhere. Therefore, the small value of the fractional order *α* helps to prolong the period of time to reach the lower epidemic peak, which in turn helps the health system to treat the largest number of infected individuals and avoid collapse.

**Figure 9:**
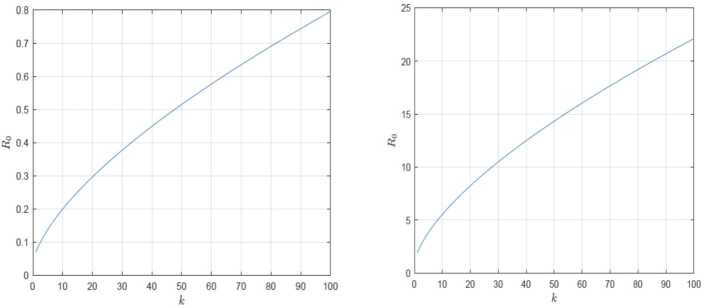
The change of $\mathcal{R}_{0}$ value with respect the value of degree *k*, the left curve for group 1 parameters and the right curve for group 2 parameters

The value of the basic reproductive number $\mathcal{R}_{0}$ depends on some parameters ($\beta _{1}$, *μ*, *B*, *γ* and $\frac{ \langle k^{2} \rangle }{ \langle k \rangle }$). The parameter $\frac{ \langle k^{2} \rangle }{ \langle k \rangle }$ is very important and representing the heterogeneity of the network. In Fig. 9 we illustrate the importance of $\frac{ \langle k^{2} \rangle }{ \langle k \rangle }$ by plotting the change of $\mathcal{R}_{0}$ value with respect the value of degree *k*.

**Figure 10:**
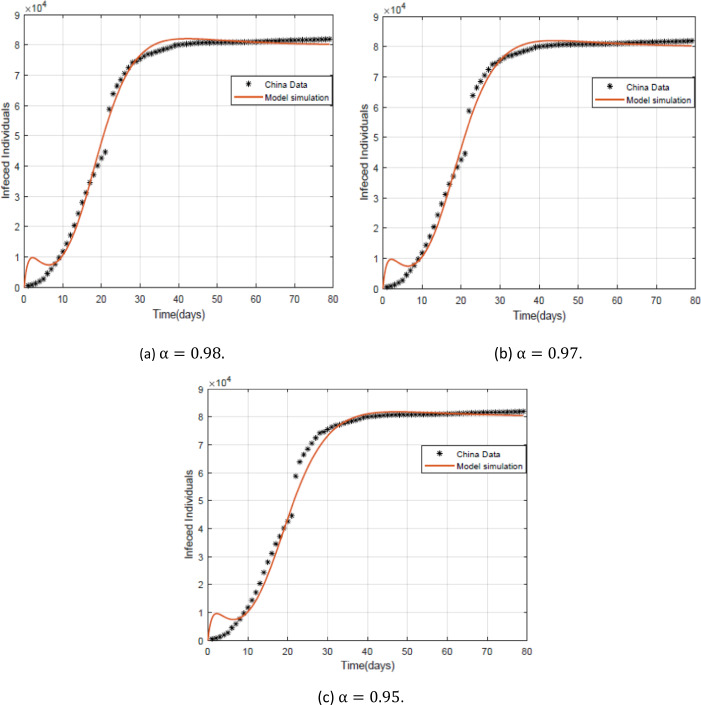
The real data of China with the prediction curve of infected persons (this curve plotted with $k=1$, $\beta _{1} = \beta _{2} =0.2$, $\mu =0.27$, $B=0.0953431859205776$, $\gamma =0. 6$ and $A= 561791$. The initial values are $S ( 0 ) = 11{,}080{,}139$, $E ( 0 ) =50{,}000$ and $I ( 0 ) =41$. We get $\mathcal{R}_{0} =2.4103$

In Fig. 10, we compare the real data [29] of China from 22 January to 9 April with the prediction curve of the infected individuals. We get the more suitable case with fractional order $\alpha =0.98$.

## Conclusion and discussion

In this paper we presented a heterogeneous epidemiological network model that illustrates the novel coronavirus (COVID-19) prevalence pattern using a fractional-order system. Taking into account the effect of the zoonotic source origin of the disease, as well as the continuous transport movement in Wuhan, the mainland city of the virus. We calculated the basic reproduction number, which significantly depends on traveling and movement rates from and outside the city. In addition we calculated the equilibrium positions for this system, as well as showing the local stability of the disease-free situation if the value of the $\mathcal{R}_{0} <1$. Likewise, the epidemiological situation is locally asymptotically stable, if the value of $\mathcal{R}_{0} >1$. And the danger of this virus (COVID-19) appears in the speed of its spread among individuals and the danger of its transmission to many countries around the world. There is a great fear of the formation of (COVID-19) for another large infection area outside the mainland and containing another strain of the corona family. We cannot deny the effective influence of the zoonotic source through which the virus was transmitted to humans and which in turn has spread among humans. It is possible that the impact of the zoonotic source continues until now and is not limited to the closure of the seafood market in Wuhan, China, which is considered as a possible explanation for the increasing numbers of infection.

## Data Availability

Not applicable.
